# OUTCOMES OF THE ADULT DAY SERVICE PLUS (ADS PLUS) PROGRAM: A HYBRID TYPE I MULTISITE TRIAL DESIGN

**DOI:** 10.1093/geroni/igad104.0522

**Published:** 2023-12-21

**Authors:** Laura Gitlin, David Roth, Katherine Marx, Lauren Parker, Sokha Koeuth, Holly Dabelko-Schoeny, Keith Anderson, Joseph Gaugler

**Affiliations:** Drexel University, Philadelphia, Pennsylvania, United States; Johns Hopkins University, Baltimore, Maryland, United States; Johns Hopkins University, Baltimore, Maryland, United States; Johns Hopkins University, Baltimore, Maryland, United States; Drexel University, Philadelphia, Pennsylvania, United States; The Ohio State University, Columbus, Ohio, United States; University of Mississippi, Oxford, Mississippi, United States; University of Minnesota, Minneapolis, Minnesota, United States

## Abstract

This paper presents the main outcomes of a cluster randomized hybrid trial (type I) involving 34 adult day services (ADS) in the US. Eighteen sites were randomized to control (ADS as usual); 16 sites provided ADS and caregiver support (ADS Plus). Trained staff met with caregivers onsite to provide dementia education, support/validation, referrals/linkages, and strategies for care challenges and self-care over 12-months. Main outcomes included depressive symptoms (CES-D) and well-being at 6-and 12-months, and attendance of clients (people living with dementia) over 12-months. Of 203 caregivers (Intervention=102; Control=101), 5.9% at 3-months, 12.8% at 6-months, and 22.7% at 12-months were lost-to-follow-up. Caregivers were predominantly female (80.3%), with 76.4% identifying as white/Caucasian, 14.8% Black/African American, and 12.3% Hispanic/Latino. Most (88.2%) had >college education and were 65.0 years old (SD=13.46). For those with 6-month data, 40.4% control and 40.2% ADS Plus caregivers had depressed symptoms (>16 CES-D) at baseline. By 6-months, 43.6% control versus 34.2% ADS Plus caregivers had >16 scores (odds ratio=0.38, p=0.072). By 12-months, after covariate adjustments, ADS Plus caregivers reported reduced total depression scores versus controls (p=0.013) and lower depressed affect scores (p=0.015). Of 18 sites providing 12-month client attendance data, nine intervention sites reported 126.05 days attended versus 78.49 days for nine control sites (p=0.079). Compared to ADS alone, by 12-months ADS Plus improved caregiver mood and increased ADS utilization by 60.6%. Results support ADS staff delivering evidence-based caregiver support to enhance benefits for clients, caregivers and ADS.

